# No Evidence for Ionotropic Pheromone Transduction in the Hawkmoth *Manduca sexta*

**DOI:** 10.1371/journal.pone.0166060

**Published:** 2016-11-09

**Authors:** Andreas Nolte, Petra Gawalek, Sarah Koerte, HongYing Wei, Robin Schumann, Achim Werckenthin, Jürgen Krieger, Monika Stengl

**Affiliations:** 1 Department of Animal Physiology, FB 10 Biology, University of Kassel, Heinrich-Plett-Str. 40, 34132, Kassel, Germany; 2 Department of Animal Physiology, Institute of Biology/Zoology, Martin-Luther-University Halle-Wittenberg, Hoher Weg 8, 06108, Halle (Saale), Germany; Plant and Food Research, NEW ZEALAND

## Abstract

Insect odorant receptors (ORs) are 7-transmembrane receptors with inverse membrane topology. They associate with the conserved ion channel Orco. As chaperon, Orco maintains ORs in cilia and, as pacemaker channel, Orco controls spontaneous activity in olfactory receptor neurons. Odorant binding to ORs opens OR-Orco receptor ion channel complexes in heterologous expression systems. It is unknown, whether this also occurs *in vivo*. As an alternative to this ionotropic transduction, experimental evidence is accumulating for metabotropic odor transduction, implicating that insect ORs couple to G-proteins. Resulting second messengers gate various ion channels. They generate the sensillum potential that elicits phasic-tonic action potentials (APs) followed by late, long-lasting pheromone responses. Because it is still unclear how and when Orco opens after odor-OR-binding, we used tip recordings to examine *in vivo* the effects of the Orco antagonist OLC15 and the amilorides MIA and HMA on bombykal transduction in the hawkmoth *Manduca sexta*. In contrast to OLC15 both amilorides decreased the pheromone-dependent sensillum potential amplitude and the frequency of the phasic AP response. Instead, OLC15 decreased spontaneous activity, increased latencies of phasic-, and decreased frequencies of late, long-lasting pheromone responses Zeitgebertime-dependently. Our results suggest no involvement for Orco in the primary transduction events, in contrast to amiloride-sensitive channels. Instead of an odor-gated ionotropic receptor, Orco rather acts as a voltage- and apparently second messenger-gated pacemaker channel controlling the membrane potential and hence threshold and kinetics of the pheromone response.

## Introduction

**Fig 1:**
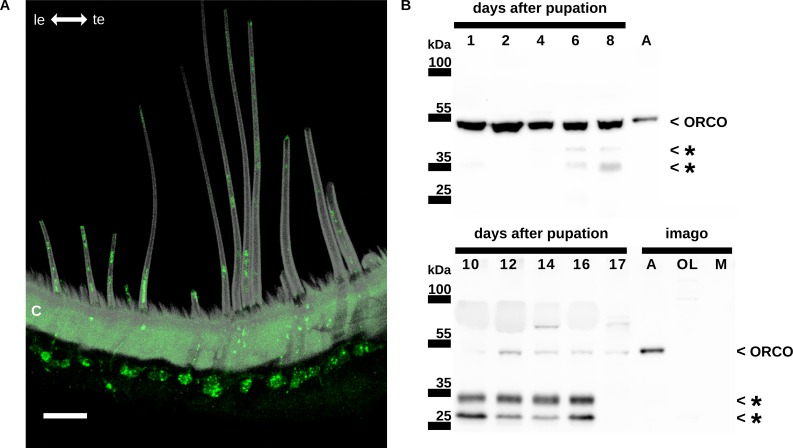
The conserved ion channel Orco is present in developing and adult hawkmoth antennae. (A) Antisera against moth Orco stain both somata of olfactory receptor neurons (ORNs) under long pheromone-sensitive trichoid sensilla in adult male antennae of the hawkmoth *Manduca sexta*. Dendrites and cilia, which extend into the cuticular hairs of the sensilla, are Orco-immunoreactive (green), while axons of the ORNs are not stained. A 27 μm maximum projection of a confocal image is shown. Grey: autofluorescence of the cuticle. Scalebar: 20 μm. C, cuticle of the antenna; le, leading edge of the antenna; te, trailing edge of the antenna. (B) Antisera against moth Orco recognize a protein of the approximate predicted molecular mass of Orco (~54 kDa). Western blots of pupal male *M*. *sexta* antennae at different days after pupation (animals eclose at pupal stage 18) show that Orco is strongly expressed at early pupal stages 1–8 and in the adult antenna. However, Orco-immunoreactive protein is hardly detectable at pupal stages 10–17 while two lower molecular weight bands are recognized by the anti-Orco antiserum (stars). We do not know, whether these lower molecular weight proteins are splice variants, degradation products of Orco, or unrelated proteins. As a control, tissues from optic lobes and muscles were used, which show no signal near the expected molecular weight of MsexOrco. A adult male antennae, kDa kilodalton, M muscle tissue of adult males; OL optic lobes.

The sense of smell is highly developed in insects. Sensitive detection of intermittent odor stimuli such as sex-pheromones is essential for mating success in various insects. Despite its general importance, insect odor transduction is still not fully understood because contradicting evidence either support ionotropic and/or metabotropic mechanisms even in the same species (reviews: [1, 2]). Ionotropic receptors are ion channels that are directly gated by their specific ligand, while metabotropic receptors are 7-transmembrane (7-TM) receptors that couple to trimeric G-proteins. They modulate the activity of enzymes that generate/deplete second messengers such as cAMP or Ca^2+^. Insect odorant receptors (ORs) are 7-TM receptors with an inverse topology resulting in an intracellular N-terminus [3–6]. One to three ORs are expressed in each insect olfactory receptor neuron (ORN) together with a conserved ubiquitous ion channel termed Orco (olfactory receptor coreceptor) [6–12]. There is general agreement that ORs but not Orco specifically bind odor ligands [11–14]. Nevertheless, Orco is essential for odor detection because it locates and maintains ORs to the membrane of ORNs [3, 15, 16]. Next to this “chaperon” function, it is generally accepted that Orco forms a spontaneously opening Ca^2+^-permeable unspecific cation channel in heterologous expression systems [17–19]. Orco is located in membranes of dendritic cilia and soma (Fig 1) and acts as a pacemaker channel controlling spontaneous activity of insect ORNs [6, 15, 19, 20]. An ion channel is termed “pacemaker channel” if it opens at the cell´s negative resting potential, driving a neuron from rest to spike threshold, thus generating spontaneous activity. In contrast to its roles as chaperon and pacemaker channel, Orco´s precise role in insect odor transduction has not been elucidated. Odorant-dependent gating of OR-Orco receptor ion channel complexes in fruitfly, mosquito, and moths studied in heterologous expression systems was taken as evidence for an exclusively ionotropic transduction mechanism in all insect species [1, 17]. Other groups suggested mixed ionotropic and metabotropic odor transduction for fruitflies [18, 21, 22]. They found that in addition to OR-Orco-dependent odor-gated currents, odors elevated cAMP levels that opened Orco after protein kinase C (PKC)-dependent phosphorylation. Finally, evidence for exclusively metabotropic signal transduction with ORs coupling to G_q_-proteins which activate phospholipase Cβ were documented for the hawkmoth *Manduca sexta* [2, 6, 19].

The identification of compounds either activating or inhibiting the highly conserved Orco as suitable target for pest control greatly facilitated further analysis of insect odor transduction. First, VUAA1 (N-(4-ethylphenyl)-2-((4-ethyl-5-(3-pyridinyl)-4H-1,2,4-triazol-3-yl)thiol)acetamide) was identified as an activator of Orco [23]. Then, the structurally related OLC15 (N-(4-butylphenyl)-2-((4-ethyl-5-(2-pyridinyl)-4H-1,2,4-triazol-3-yl)thio)acetamide) was identified as the most potent antagonist of Orco in different insect species [24]. Furthermore, amiloride derivatives (HMA, MIA, see Material and Methods) that block odor transduction in crustaceans [25] were suggested to also block insect odorant receptor currents [26, 27].

To clarify the role of Orco in insect odor transduction we chose the nocturnal hawkmoth *M*. *sexta* as well established model for insect olfaction [28, 29]. Previously, we showed that application of the Orco agonist VUAA1 in tip-recordings from pheromone-sensitive trichoid sensilla of hawkmoth antennae increased spontaneous activity and the late, long-lasting pheromone response, which occurs seconds to minutes after pheromone application. However, VUAA1 did not affect parameters of the pheromone response within the first ~100 ms of bombykal-responses, making ionotropic transduction unlikely [19]. Here, we further challenged the hypothesis of Orco-dependent ionotropic odor transduction with the Orco antagonist OLC15 and the amiloride derivatives HMA and MIA in tip-recordings of bombykal-sensitive trichoid sensilla on hawkmoth antennae. In agreement with previous results we found no evidence for Orco-based ionotropic pheromone transduction in *M*. *sexta*, but provide evidence for a function of Orco as voltage- and second messenger-gated pacemaker channel that controls the membrane potential of ORNs.

## Material and Methods

### Animals and preparation

All experiments were performed on adult or pupal male *M*. *sexta*, raised from egg in the rearing facilities of the University of Kassel as described previously [30]. Animals were entrained to a 17:7h light:dark cycle with Zeitgebertime (ZT) 0 light on and ZT 17 light off. In our cultures the nocturnal hawkmoths are still active at ZT 1–3 before becoming inactive during the day. Thus, to test for activity- but not illumination-dependent effects, recordings were performed at room temperature (19–23°C) in the animal’s activity phase (ZT 1–3) and during rest (ZT 9–11).

### Tip-recordings and pharmacology

The 1–2 day old male moths were fixed in a custom made Teflon holder by adhesive tape with the antenna additionally fixed by dental wax prior to recordings. The apical segments of the fixed antenna were removed, and the indifferent glass electrode filled with hemolymph ringer (6.4 mM KCL, 12.0 mM MgCl_2_×6H_2_O, 1 mM CaCl_2_×2H_2_O, 12.0 mM NaCl, 10.0 mM HEPES, 354.0 mM glucose-monohydrate, pH 6.5, osmolarity: 450 mOsmol/l) was inserted. Tips of trichoid sensilla on the second remaining antennal segment were cut, and the recording electrode filled with sensillum lymph ringer (171.9 mM KCL, 3.0 mM MgCl_2_×6H_2_O, 1 mM CaCl_2_×2H_2_O, 25.0 mM NaCl, 10.0 mM HEPES, 22.5 mM glucose-monohydrate, pH 6.5, osmolarity: 475 mOsmol/l) was slipped over one sensillum. All pharmaceuticals used were diluted with dimethyl sulfoxide (DMSO) in sensillum lymph ringer to a concentration of 10 μmol/l containing 0.1% DMSO if not otherwise specified. 5-(N-methyl-N-isobutyl)amiloride (MIA) and 5-(N,N-hexamethylene)amiloride (HMA) were obtained from Sigma-Aldrich (Sigma-Aldrich Co., Munich, Germany). N-(4-ethylphenyl)-2((4-ethyl-5-(3-pyridinyl)-4H-1,2,4-triazol-3-yl) thio)acetamide (VUAA1) and N-(4-butylphenyl)-2-((4-ethyl-5-(2-pyridinyl)-4H-1,2,4-triazol-3-yl)thio)acetamide (OLC15) were generously provided by the Max-Planck-Institute for Chemical Ecology, Jena. Three different concentrations (1,10, and 100 μM) were employed for VUAA1 and OLC15. Agents diffused passively via the recording electrode into the sensillum. For stimulation with the main pheromone component bombykal (BAL, 1 μg on filter paper) a non-adapting stimulus protocol (50 ms pulse duration, 5 min interstimulus intervals) was employed as described previously [19]. The pheromone concentration employed was not saturating but was in the middle range of the dose-response curve [31]. Each recording lasted 20 minutes with 5 consecutive BAL stimulations, separated by 5 min each. One experiment contained 10 recordings with a total of 50 stimulations. Four different experiments were conducted at both ZT-times investigated: 1. controls (only containing 0.1% DMSO), 2. application of 10 μM MIA, 3. application of 10 μM HMA, and 4. application of 10 μM OLC15.

### Current injection

To depolarize ORNs without pheromone application, a current was injected via the recording electrode. The stimulation unit of the amplifier (BA-03x, npi Electronic, Tamm, Germany) was connected to a personal computer via TTL link. Current injection of 3 nA for 10 s reliably increased depolarization-dependent neuronal activity without causing long-term damage. Neuronal activity of the ORN was measured for 60 s after current injection and compared to the spontaneous activity before current injection.

### Data acquisition and analysis

Recordings were performed using Clampex 8 software (Molecular Devices, Sunnyvale, CA, USA). Neuronal activity was recorded gap-free for 5 s after each pheromone stimulation (sampling rate: 20 kHz). Spike2 software (version 7.01; Cambridge Electronic Design, Cambridge, UK) was used to analyse parameters of the initial pheromone response: normalized sensillum potential amplitude (SPA), frequency of the first 10 BAL-dependent action potentials (APs) and latency of the first AP to the onset of the SPA. To analyse the phasic-tonic response pattern of ORNs the distribution of APs in the first 600 ms after pheromone stimulation was plotted in post-stimulus time histograms (PSTHs, binwidth: 10 ms). For the remaining 295 s (until the next stimulation), APs were recorded as fixed length events (sampling rate: 19.6 kHz, length of recorded events: 12.75 ms). These APs had to pass a trigger, which was set to about one third of the maximum height of an average AP in the highpass filtered AC trace. The number of APs between two BAL-stimuli was analyzed as the late, long-lasting pheromone response which is characterized by an increased neuronal activity seconds to minutes after stimulation. Spontaneous activity and activity induced by current injection were recorded for 60 s as triggered events. Values of the parameters of each experimental approach were plotted in a graph over time showing mean ± standard error for each stimulation. For statistical analysis the values of all 5 stimulations in each recording were summed according to the experimental approach and shown in box plots because values showed a non-Gaussian distribution (box: 50% quantile and median, whiskers: 5 to 95 percentiles).

### Statistics

Statistical analysis was performed using GraphPad Prism 6.01 (GraphPad Software, La Jolla, CA, USA). Data were first analysed for Gaussian distribution using Shapiro-Wilk normality test. Because the data did not show Gaussian distribution the Mann-Whitney test was employed for comparing two groups, and the Kruskal-Wallis test with Dunn’s post-hoc test was used for mulitple comparison (confidence interval α = 0.05). All figures were prepared using GraphPad Prism and Corel Draw X3 (Corel Corporation, Ottawa, Canada). According to the P values significant and non-significant differences were shown in figures as asterisks or n.s. (n.s. = not significant; *P<0.05; **P<0.01; ***P<0.001).

### Immunocytochemistry

The polyclonal anti-Orco antibody was raised against the peptide NH_2_-NQSNSHPLFTESDARYH-COOH, which is identical in *Bombyx mori*, *Heliothis virescens*, and *M*. *sexta*. The peptide was conjugated to KLH (Squarix Biotechnology, Marl, Germany), and standard procedures were applied to immunize rabbits (Charles River Laboratories, Kisslegg, Germany). Specificity was confirmed with preadsorption against its respective peptide-epitope. In addition, omission of the primary antibodies tested for unspecific staining. Antibodies were purified by peptide-affinity chromatography (Squarix Biotechnology) to a final concentration of 1.74 μg/μl and used in a dilution of 1:1000 for Western blots and immunostainings. Male *M*. *sexta* antennae were cut into 5–10 annuli each, fixed for 2 h in phosphate buffered saline containing 4% formaldehyde (Roti Histofix, Roth, Karlsruhe, Germany, pH 7), 10% (w/v) sucrose and 1% (v/v) Triton X-100. After fixation the antennal fragments were washed 3x5 min with 0.1 M sodium phosphate buffer (PB), pH 7.4, containing 1% (v/v) Triton X-100 and subsequently incubated in 0.1 M PB containing 25% (w/v) sucrose at 4°C over night. After cryoprotection, fragments were washed, embedded into Jung tissue freezing medium (Leica, Wetzlar, Germany), sectioned at 30 μm with a cryostat-microtome (Leica CM3050) and transferred to Polylysin slides (Thermo Scientific, Waltham, MA). After sections had dried at room temperature, slides were washed 3x5 min with 0.1 M PB containing 0.5% (v/v) Triton X-100 and preincubated in PBS (10 mM Na_2_HPO_2_, 2 mM KH_2_PO_2_, 2.7 mM KCl, 137 mM NaCl) containing 10% (v/v) normal goat serum (NGS, Dianova, Hamburg, Germany) and 0.3% (v/v) Triton X-100 (blocking solution). Primary antibody incubation was performed over night at 4°C in blocking solution. Slides were then washed 3x5 min with 0.1 M PB containing 0.1% (v/v) Triton X-100 and incubated for 2 h at room temperature in blocking solution containing Cy3-coupled goat anti-rabbit antibodies (Dianova) at a 1:300 dilution. After washing 3x5 min in 0.1 M PB, slides were dehydrated in an increasing ethanol series and embedded in Entellan (Merck, Darmstadt, Germany). Sections were scanned with a confocal laser-scanning microscope (Leica TCS SP5) at a resolution of 264 nm/square pixel, with a z-step size of 629 nm, using a Leica HCX PL apochromate 20X/0.7 multi-immersion objective. The Cy3 fluorescent dye was excited at 543 nm and detected between 555 nm and 625 nm, autofluorescence of the cuticle was excited at 633 nm and detected between 650 nm and 730 nm. The Leica image file was imported autoscaled to ImageJ 1.5b using the Bioformats Plugins, the stack was cropped, and the maximum projection was calculated using the maximum intensity projection type. Brightness and contrast were adjusted for each channel image-wide.

### Western blots

Four antennae of two animals were homogenized in RIPA buffer (50 mM TrisHCl, 30 mM NaCl, 1% (v/v) IGEPAL CA-360, 1% (w/v) sodium cholate hydrate, 0.1% (w/v) SDS), incubated for 30 min at 60°C and centrifuged twice for 5 min, 17000 g to remove cuticle and cell debris. The homogenate was then diluted 1:2 with sample buffer (500 mM Tris, 5% (v/v) glycerol, 1.5 mM bromphenoleblue, 5% (v/v) β-mercaptoethanol, 0.25% (w/v) SDS; pH 6.8) and 10 μl of each sample were loaded on a 10% polyacrylamide gel (Mini-PROTEAN 3, Biorad, Hercules, CA). Proteins were blotted for 1 h at 100 V (constant, max. 330 mA) to PVDF membrane. After blotting, the membrane was blocked for 1 h at room temperature with TBS (20 mM TrisHCl, 140 mM NaCl; pH 7.5) containing 5% (w/v) milk-powder and 0.1% (v/v) Tween 20 and thereafter incubated over night at 4°C with the anti-ORCO antibody in TBS containing 0.1% (v/v) Tween 20 and 1% (w/v) milk-powder. It was subsequently washed for 3x1 min, 1x5 min, 1x10 min and 1x30 min with TBS containing 0.05% (v/v) Tween 20, 1% (v/v) Triton X-100 and 0.1% (w/v) SDS. The blot was then incubated for 1 h at room temperature with HRP-coupled secondary goat anti-rabbit antibodies (Dianova) which were diluted 1:10,000 in TBS containing 0.1% (v/v) Tween 20 and washed as described before with two additional steps for 5 min each in TBS containing 0.05% (v/v) Tween 20. The chemiluminescent signal was recorded with a LI-COR Odyssey Fc system (LI-COR Biosciences, Lincoln, NE).

### Primary cell culture

Primary cell cultures were performed according to published protocols [32, 33]. Briefly, antennal flagella were removed from 2 day-old pupae (eclosion: 18 day-old pupae) and were disrupted with mechanical and enzymatic treatment. The dissociated cells were grown on concanavalin-A-coated cover slips, in Leibowitz (L15) medium supplemented with 5% fetal bovine serum (Gibco) and conditioned medium of a non-neuronal *M*. *sexta* cell line (Dwight Lynn). The cultures (n>20) were grown at 20°C and high humidity for about two weeks before analysis with Ca^2+^ imaging. Antennal cultures contain many different antennal cell types. While sensory receptor neurons could be recognized according to morphological criteria, OR-expressing or pheromone receptor-expressing ORNs could not be recognized in the diverse cultures, based upon morphological criteria alone [32, 33].

### Ca^2+^ imaging

The experiments were performed according to published protocols [34]. Briefly, primary cell cultures of ORNs, which reached a mature, pheromone-sensitive developmental stage [32, 35], were loaded with 1 μM Ca^2+^ indicator Fura-2 acetoxymethyl ester (Fura-2 AM, Molecular Probes, Eugene, OR, USA) for 40 min at room temperature. After loading, the cover slip with the primary cell cultures of ORNs was transferred to a perfusion chamber on the stage of an Examiner D1 microscope (Zeiss, Germany), equipped with a CCD camera (Andor 885, Andor Technology, N-Ireland). Images were acquired with Tillvision 4.0 software (Till-Photonics, Gräfelfing, Germany). Excitation wavelengths for Fura-2 were 340 nm (exposure time, 100 ms) and 380 nm (exposure time 50 ms) with 600 ms intervals. The dissociation constant for Fura-2 was calculated with calibration solutions and linear regression analyses (Origin 6.0) [34]. VUAA1 was applied at different concentrations via perfusion (REGLO Digital MS-2/6, Ismatec, IDEX Health & Science, Germany) at 1ml/min flow rate. Perfusion was applied continuously with normal standard saline solution (mM): 156 NaCl, 4 KCl, 2 CaCl_2_, 10 hepes, 5 glucose, pH 7.1, 380 mOsm/kg. After finding three ORNs in the diverse cultures (n>20) that expressed VUAA1-dependent Ca^2+^ rises dose-dependently, these costly, time-consuming experiments were stopped.

## Results

**Fig 2:**
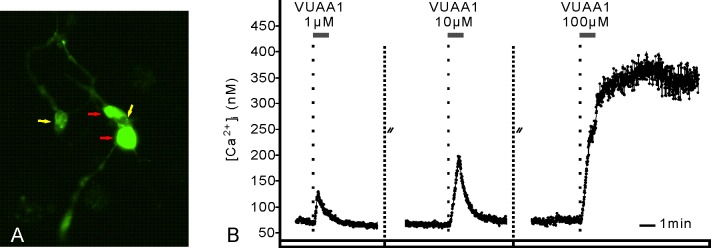
Orco agonist VUAA1 activates MsexOrco in olfactory receptor neurons (ORNs) of hawkmoths. (A) Two weeks-old primary cell cultures of pupal antennae contain two antennal cells strongly filled with Fura-2 (red arrows), attached to less-stained non-neuronal cells (yellow arrows) with long processes. According to its soma size of about 10 μm and its fine processes (not stained) the smaller of the two strongly stained cells is an ORN. (B) In Ca^2+^ imaging studies VUAA1 dose-dependently (1 μM, 10 μM, 100 μM) increased intracellular Ca^2+^ levels in the small ORN shown in A, identifying it as an Orco-expressing ORN. Primary cell cultures of ORNs from 2 day-old pupae were grown for two weeks *in vitro* before stimulation. At this stage the ORNs matured and were shown previously to become pheromone-responsive [35].

So far hypotheses of Orco function in insect odor transduction were based upon studies in heterologous expression systems (review: [6]).To examine the role of *M*. *sexta* Orco (MsexOrco) in pheromone transduction *in vivo*, we first confirmed with antisera against moth Orco that Orco is present in pheromone-sensitive trichoid sensilla of male hawkmoth antennae. As predicted for a chaperon function and a pacemaker function of Orco, Orco-antibodies stained the sensory cilia, dendrites, and somata, but not the axons of pheromone-specific ORNs in cryostat sections of adult *M*. *sexta* male antennae (Fig 1A). Previously, we demonstrated in Ca^2+^ imaging experiments that Orco activator VUAA1 can activate MsexOrco in heterologous expression systems [19]. Now we examined with Ca^2+^ imaging, whether Orco agonist VUAA1 activates Orco also in its natural *in vivo* environment, in pheromone-sensitive ORNs of the hawkmoth. Thus, we tested, whether VUAA1 can also increase intracellular Ca^2+^ levels in primary cell cultures of *M*. *sexta* ORNs at a stage, when they respond to pheromones *in vitro* [32, 35]. Western blots demonstrated that the Orco-antiserum recognized a protein with the expected mass of Orco in homogenates of the adult antenna of male hawkmoth (Fig 1B). In addition, Orco is already present at early stages of the developing pupal antenna even before ORNs respond to pheromones [36] (Fig 1B). Orco is present throughout larval development, but apparently at varying concentrations. We obtained primary cell cultures of ORNs from male antennae at pupal day 2, when ORNs were just born. The primary cultures were grown for about two weeks *in vitro*. At this stage they were mature and responded to pheromones, as shown previously [32, 35]. Application of the Orco agonist VUAA1 increased intracellular Ca^2+^ concentrations dose-dependently in these primary cell cultures of matured hawkmoth ORNs, similar to its effect in heterologous expression (n = 3) (Fig 2). Thus, the Orco agonist VUAA1 recognizes and activates MsexOrco not only in heterologous expression, but also in its natural environment in hawkmoth ORNs.

**Fig 3:**
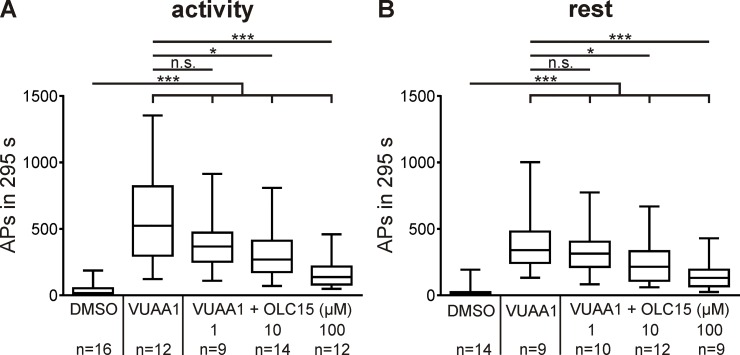
Orco antagonist OLC15 blocks *Manduca sexta* Orco *in vivo*. (A,B) In tip-recordings of pheromone-sensitive trichoid sensilla in intact hawkmoths, Orco agonist VUAA1 (10 μM) increased spontaneous activity of unstimulated, pheromone-sensitive olfactory receptor neurons, as compared to controls (DMSO). During the activity phase (A) application of VUAA1 was significantly more effective than at rest (B) (Table 1). Co-application of OLC15 dose-dependently (1, 10, 100 μM) interferes with VUAA1-dependent activation of Orco. About half of the ORNs maximal activity was blocked with an intermediate concentration of 10 μM OLC15. Box plots with whiskers from 5 to 95 percentiles. Significant differences are indicated by asterisks (for exact P-values see Table 1; n.s. = not significant; **P<0.01; ***P<0.001).

Next, we confirmed that VUAA1 activates MsexOrco also *in vivo* in pheromone-sensitive ORNs of trichoid sensilla in intact hawkmoths [19]. Then, we examined, whether OLC15 is an inhibitor of MsexOrco that can interfere with VUAA1-dependent activation of MsexOrco in adult antennae (Fig 3). There is general agreement that the highly conserved Orco affects spontaneous activity of ORNs in different insect species and, thus, acts as a pacemaker channel (review: [6]). If VUAA1 is an activator of MsexOrco in adult antennae, it should, therefore, increase spontaneous activity of pheromone-sensitive, adult ORNs. Furthermore, if OLC15 is an inhibitor of MsexOrco, it should dose-dependently interfere with VUAA1-dependent activation. Tip-recordings were performed from long, pheromone-sensitive trichoid sensilla on the antennae of adult male *M*. *sexta*. Each trichoid sensillum is innervated by two ORNs, one of which responds to bombykal, the main sex-pheromone component of the female hawkmoth. Because previous analyses revealed Zeitgebertime (ZT)-dependent changes of pheromone transduction (review: [2]), experiments were performed at the end of the moth´s activity phase (ZT 1–3) and during its resting phase (ZT 9–11) (Material and Methods).

Spontaneous activity of ORNs in adult antennae usually is very low. Thus, activity was increased via infusion of the Orco agonist VUAA1 [19, 23] and compared to recordings with co-application of three different doses of OLC15. Orco agonist VUAA1 increased “spontaneous” activity more effectively during the moth`s activity phase than during the resting phase, pointing to a lower level of active MsexOrco at rest (Fig 3A and 3B; Table 1). Co-infusion of OLC15 dose-dependently blocked VUAA1-dependent activity. In these experiments 10 μM OLC15 blocked about half-maximal activity at both ZTs (Fig 3). In addition, in long-term tip recordings, periods of higher spontaneous activity of pheromone-sensitive trichoid sensilla were determined. It was confirmed that also in the absence of VUAA1, OLC15 blocks spontaneous activity (not shown). Thus, OLC15 blocks MsexOrco not only in heterologous expression but also *in vivo* in adult pheromone-sensitive trichoid sensilla. Consequently, we employed OLC15 as MsexOrco antagonist in our further experiments at its intermediate concentration of 10 μM.

**Table 1:** Statistics for OLC15-dependent inhibition of VUAA1-induced activity of olfactory receptor neurons *in vivo*.

*P<0.05; **P<0.01; ***P<0.001	P-value			
	Activity	Rest		Activity–Rest
DMSO–VUAA1 (10 μM)	<0.0001	<0.0001	DMSO	0.0136
DMSO–VUAA1+OLC15 (1 μM)	<0.0001	<0.0001		
DMSO–VUAA1+OLC15 (10 μM)	<0.0001	<0.0001	VUAA1 (10 μM)	0.0102
DMSO–VUAA1+OLC15 (100 μM)	<0.0001	0.0004		
VUAA1 –VUAA1+OLC15 (1 μM)	>0.9999	>0.9999	VUAA1+OLC15 (1 μM)	0.1046
VUAA1 –VUAA1+OLC15 (10 μM)	0.0260	0.0144		
VUAA1 –VUAA1+OLC15 (100 μM)	<0.0001	<0.0001	VUAA1+OLC15 (10 μM)	0.0320
VUAA1+OLC15 (1 μM)–VUAA1+OLC15 (10 μM)	>0.9999	0.0972		
VUAA1+OLC15 (1 μM)–VUAA1+OLC15 (100 μM)	<0.0001	<0.0001	VUAA1+ OLC15 (100 μM)	0.4102
VUAA1+OLC15 (10 μM)–VUAA1+OLC15 (100 μM)	0.0018	0.0071		

Kruskal-Wallis- with Dunn’s tests for multiple comparison of all experiments at one Zeitgebertime were employed, α = 0.05. Comparison of each respective experiment during the activity phase with the rest phase was accomplished with the Mann-Whitney test, α = 0.05.

**Fig 4:**
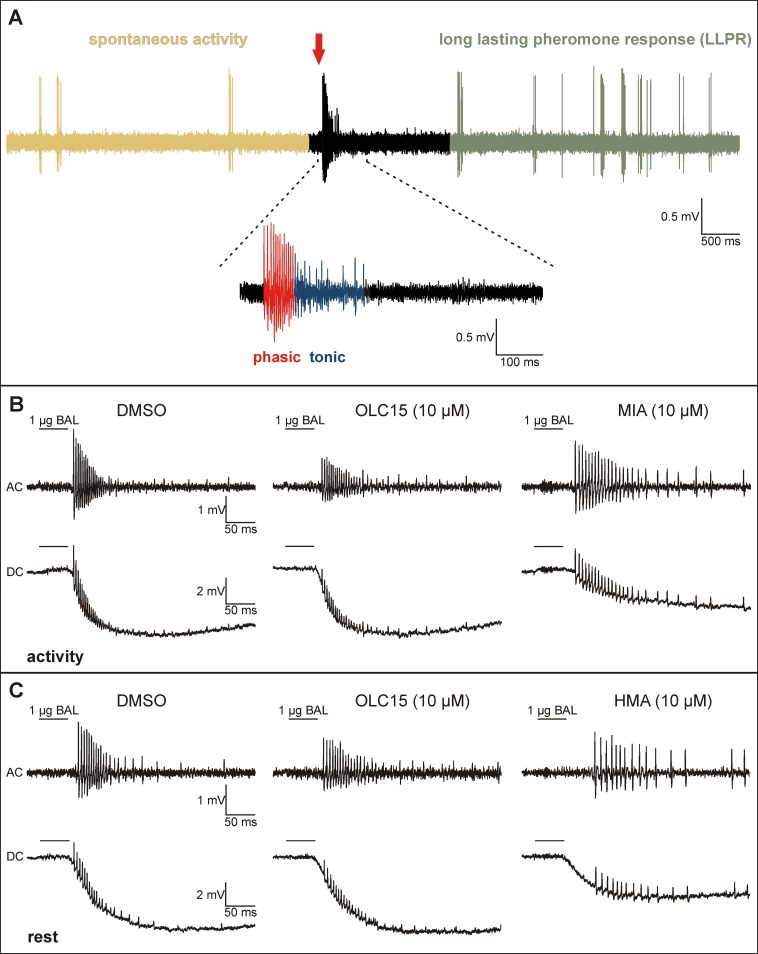
OLC15, MIA, and HMA affect different targets of the pheromone transduction cascade. (A) The bombykal (50 ms; 1 μg on filter paper)—dependent AP response consists of at least 3 phases which occur at characteristic time windows after the pheromone stimulus (red arrow), also at other pheromone doses employed. In the absence of pheromone, olfactory receptor neurons (ORNs) show low rates of spontaneous activity (yellow). ORNs respond to pheromone with a fast, phasic (red), and a delayed, tonic (blue) series of APs (enlargement). After a period of inhibition the late, long-lasting pheromone response (LLPR, green) begins and can last for several seconds up to minutes. (B, C) In contrast to both amilorides, Orco antagonist OLC15 does not affect the primary, phasic, or the tonic pheromone response in tip-recordings of intact hawkmoth antennae, during activity- (B) and rest-phase (C). The bombykal-dependent sensillum potential (DC) and phasic action potential response (AC) were not affected by OLC15 (10 μM), as compared to controls (DMSO) (B, C). In contrast, both parameters of the primary pheromone response were decreased Zeitgebertimer (ZT)-dependently by MIA (10 μM; shown only at activity (B)) and HMA (10 μM; shown only at rest (C)).

We designed our further experiments to distinguish between the established pacemaker function of Orco and an additional function as OR-Orco receptor ion channel complex. As pacemaker channel Orco controls the membrane potential, possibly voltage- and second messenger-dependently. Thus, it controls the threshold of the pheromone response and its kinetics, since it produces membrane potential oscillations of different amplitude and frequency. Depending on the strength of the block, the pacemaker channel will hyperpolarize the cell to different extents. Very strong hyperpolarization via full block of the channel could move it far away from spike threshold, which cannot be compensated for by odor-dependent depolarizations. Thus, a full block of a pacemaker channel will affect odor responses and the results cannot distinguish between a function of Orco as pacemaker channel or as ionotropic receptor. When the pacemaker channel is not fully blocked (around its IC_50_ value, at intermediate concentrations of antagonists), it will hyperpolarize the cell to a lesser extent, and the odor-dependent depolarization can reach spike threshold. Thus, a partial block would neither affect the receptor potential, nor the frequency of the phasic pheromone-response (Fig 4A). In contrast, an Orco-dependent ionotropic transduction mechanism would strongly affect both, the pheromone-dependent receptor potential, and the phasic pheromone response. Binding of bombykal would gate a specific OR-Orco receptor ion channel complex, and the influx of cations via OR-Orco would generate the depolarizing receptor (sensillum) potential. Therefore, block of Orco also at intermediate concentrations of antagonists would decrease the bombykal-dependent sensillum potential.

**Fig 5:**
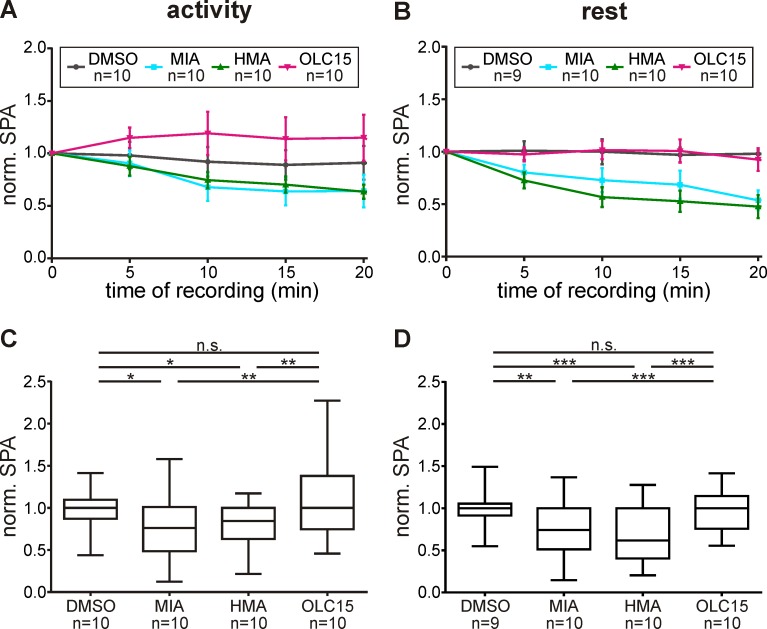
In contrast to amilorides OLC15 does not affect the pheromone-dependent normalized sensillum potential amplitude (SPA). (A,B) In controls (DMSO) bombykal (1 μg)-dependent SPAs remained constant during 20 min tip-recordings at both activity- (A,C) and rest-phase (B,D). While OLC15 did not significantly affect SPAs at both Zeitgebertimes, both amilorides, HMA and MIA, decreased the SPA. (A,B) Mean ± SEM. (C,D) Box plots with whiskers from 5 to 95 percentiles. Significant differences are shown by asterisks (exact P-values see Table 2; n.s. = not significant; *P<0.05; **P<0.01; ***P<0.001).

Infusion of 10 μM OLC15 did not affect the bombykal-dependent sensillum potential response at both ZTs tested (Figs 4B, 4C and 5A–5D; Table 2). These results were consistent with a pacemaker function of Orco, but not with a function in an ionotropic transduction mechanism. Therefore, we searched for antagonists of the ion channels that are responsible for the pheromone-dependent sensillum potential. Previous patch clamp studies suggested that pheromone activates a phospholipase Cβ (PLCβ)-dependent metabotropic cascade in *M*. *sexta* ORNs. Resulting activation of different types of Ca^2+^-dependent cation channels, with properties of TRP channels, appeared to underlie the bombykal-dependent sensillum potential response [33, 35]. Amiloride application blocked Ca^2+^-dependent cation channels in moth ORNs [26]. These channels resembled the PLCβ- and pheromone-dependently activated Ca^2+^-gated cation current of hawkmoths [33, 35]. Furthermore, amilorides blocked odor transduction via interference with TRP-like ion channels in crustacean ORNs [25]. Thus, we examined whether the amiloride derivatives HMA and MIA interfere with hawkmoth pheromone transduction. Both amilorides significantly reduced the bombykal-dependent sensillum potential amplitude with HMA being more effective at rest (Figs 4B, 4C and 5A–5D; Table 2). Thus, both amilorides appeared to target ion channels which are not identical with Orco. Furthermore, in contrast to Orco, the amiloride-dependent ion channels underlie the pheromone-dependent sensillum potential ZT-dependently. It cannot be excluded that, in addition, both amilorides also affected Orco.

**Table 2:** Statistics for bombykal responses.

*P<0.05; **P<0.01; ***P<0.001	P-value							
	SPA		ISI10		Latency		LLPR	
	Activity	Rest	Activity	Rest	Activity	Rest	Activity	Rest
DMSO–HMA	0.0134	<0,0001	0.0680	<0.0001	0.0002	<0.0001	<0.0001	<0.0001
DMSO–MIA	0.0173	0.0016	0.0031	0.0525	0.0026	<0.0001	<0.0001	>0.9999
DMSO–OLC15	>0.9999	>0.9999	0.2200	0.3497	0.0021	0.0007	0.0001	>0.9999
HMA–MIA	>0.9999	>0.9999	>0.9999	0.0024	>0.9999	>0.9999	>0.9999	<0.0001
HMA–OLC15	0.0037	<0.0001	>0.9999	0.0001	>0.9999	>0.9999	>0.9999	<0.0001
MIA–OLC15	0.0048	0.0008	0.8272	>0.9999	>0.9999	>0.9999	>0.9999	>0.9999
DMSO–OLC15 (100 μM)	<0.0001	<0.0001	0.0005	<0.0001	-	-	<0.0001	<0.0001
OLC15 –OLC15 (100 μM)	0.0042	<0.0001	0.0961	0.0190	-	-	0.0271	<0.0001

For comparison of respective experiments at one Zeitgebertime, Kruskal-Wallis- with Dunn’s tests for multiple comparison were employed, α = 0.05; SPA = sensillum potential amplitude; ISI10 = interspike interval of the first 10 action potentials (APs); LLPR = late, long-lasting pheromone response. All pharmaceuticals were used at a concentration of 10 μM, if not stated otherwise.

**Fig 6:**
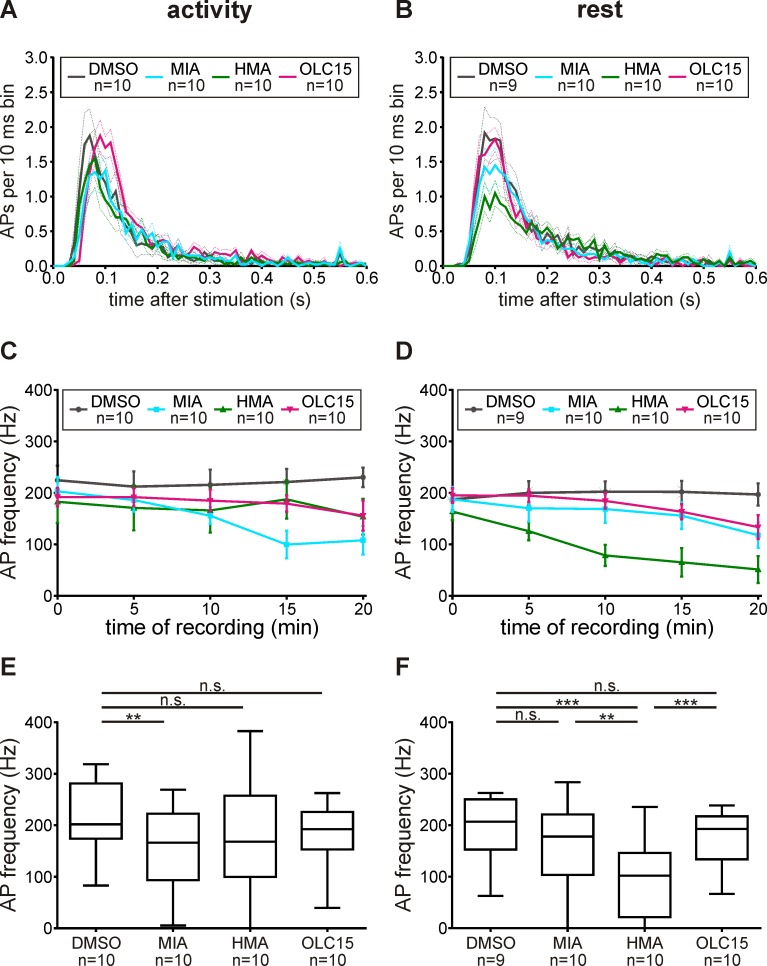
Amilorides, but not OLC15, reduced the phasic bombykal (BAL)-dependent action potential (AP) response Zeitgebertime-dependently. (A,B) Post-stimulus time histograms showing that OLC15 (magenta) did not affect the number and distribution of BAL-dependent phasic APs during activity- (A) and rest-phase (B), but significantly delayed AP response onset during the activity phase. In contrast, both amilorides decreased the phasic BAL-response, as well as the frequency of the first 10 BAL-dependent APs, with stronger effects for MIA (cyan) during activity (A,C,E), and HMA (green) during rest (B,D,F). (A,B) Bin size = 10 ms. Mean ± SEM (dotted line). (C,D) Mean ± SEM. (E,F) Box plots with whiskers from 5 to 95 percentiles. Asterisks indicate significant differences (exact P-values see Table 2; n.s. = not significant; *P<0.05; **P<0.01; ***P<0.001).

**Fig 7:**
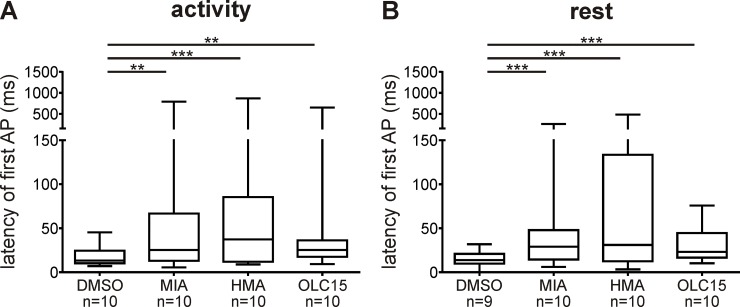
The latency of the first bombykal (BAL)-dependent AP was prolonged by all compounds tested. (A,B) The amiloride HMA was significantly more potent than OLC15 and MIA at both Zeitgebertimes. Box plots with whiskers from 5 to 95 percentiles. Different scales were used with interrupted error bars to improve illustration of differences. Significant differences are indicated by asterisks (exact P-values see Table 2; *P<0.05; **P<0.01; ***P<0.001).

In the pheromone-dependent action potential (AP) response of ORNs three characteristic phases can be distinguished. A first rapid, phasic response lasts less than 100 ms. A second slower, tonic response lasts several hundred ms. Then, after an inhibition of activity, a third very slow, late, long-lasting pheromone response can last from seconds to minutes after the pheromone stimulus (Fig 4A). An Orco-based ionotropic pheromone transduction mechanism would be expected to strongly affect the phasic AP response, while a metabotropic transduction cascade would affect either all phases of the pheromone response or, in case of a mixed ionotropic-metabotropic cascade, only the two late phases. Post-stimulus time histograms and statistical analyses of the bombykal response revealed that both amilorides decreased the frequency of the phasic AP response ZT-dependently. While MIA significantly reduced the phasic AP response only at the activity phase, HMA only reduced it during rest (Fig 6A–6F, Table 2). This confirmed that both amilorides target different ion channels of the primary transduction cascade. In contrast, Orco-antagonist OLC15 did not affect the frequency of the phasic pheromone response at either ZT. However, OLC15 specifically increased the latency of the phasic pheromone response at both ZTs being more effective during the activity phase (Fig 7A and 7B, Table 2). Also, both amilorides prolonged the latency of the bombykal responses at both ZTs (Fig 7A and 7B). These results are consistent with a pacemaker channel function of Orco, but not with a function in an ionotropic transduction mechanism. Both amilorides appeared to target different ion channels both of which were not identical to MsexOrco. The MIA-dependent ion channel was predominantly activated during the first 100 ms of the phasic pheromone response during the activity phase of the hawkmoth, at night. In contrast, the HMA-dependent ion channel was predominantly activated in the phasic pheromone response during the day, when the hawkmoth is usually resting.

**Fig 8:**
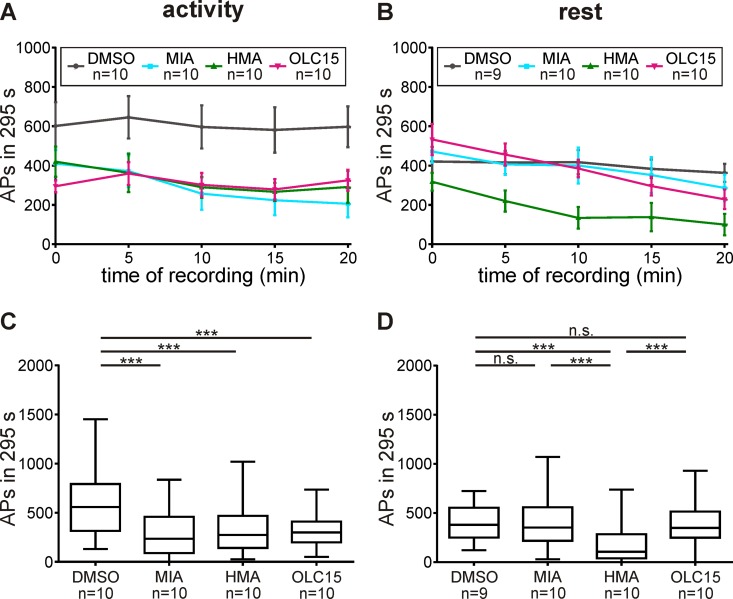
**During activity (A,C) the late, long-lasting pheromone response (LLPR) was significantly decreased by all compounds tested.** Only HMA decreased LLPR during rest (B,D). The LLPR neither includes the phasic, nor the tonic pheromone response (Fig 2C), but was measured from 5–300 s after pheromone stimulation. In control recordings (DMSO) the number of APs in the LLPR was higher during the activity-phase than during the rest-phase. (A,B) Mean ± SEM. (C,D) Box plots with whiskers from 5 to 95 percentiles. Significant differences are shown by asterisks (exact P-values see Table 2; n.s. = not significant; *P<0.05; **P<0.01; ***P<0.001).

To determine whether Orco and the amiloride-dependent ion channels might affect the late, long-lasting pheromone response, we examined their effects in a time interval ranging from 5 to 300 seconds after pheromone application (Fig 8A–8D). While HMA decreased the late, long-lasting pheromone response at both ZTs, OLC15 and MIA decreased the late, long-lasting pheromone response only during the activity phase (Fig 8A–8D, Table 2). Thus, during the first 100 ms of the pheromone response at the hawkmoth`s activity phase not Orco but only the MIA-dependent ion channel was activated. However, seconds after the pheromone stimulus Orco was activated and the MIA-dependent ion channel was also still active. In contrast, during rest the HMA-dependent ion channel dominated the first 100 ms of the pheromone response, and it remained active seconds after pheromone stimulation, while Orco and the MIA-dependent ion channel were not significantly active.

**Fig 9:**
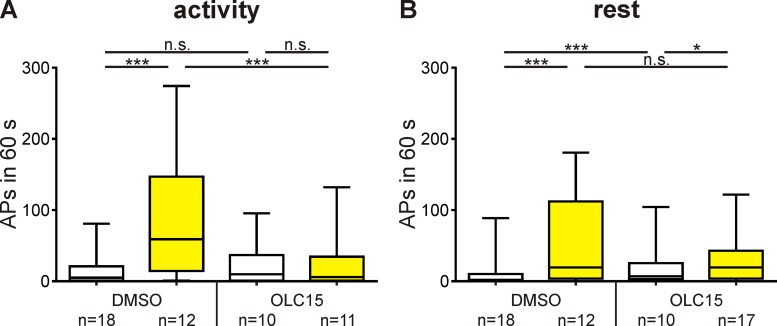
Orco is a voltage-dependent ion channel controlling spontaneous activity mainly during the hawkmoth´s activity phase. In tip-recordings of non-stimulated pheromone-sensitive trichoid sensilla the spontaneous action potential (AP) activity within 60 s after current injection (10 s, 3 nA, yellow) was increased more strongly during activity- (A) than during rest-phases (B) (Table 3). During the activity phase voltage-dependent rise in spontaneous activity was primarily mediated via Orco, since OLC15 prevented it (A). However, Orco seems to play a minor role in mediating the voltage-dependent rise in spontaneous activity during rest (B). Box plots with whiskers from 5 to 95 percentiles. Significant differences are indicated by asterisks (exact P-values see Table 3; n.s. = not significant; *P<0.05; ***P<0.001).

All data collected *in vivo* are consistent with a function of MsexOrco as a pacemaker channel, but not with a role as an OR-Orco receptor ion channel complex that underlies an ionotropic pheromone transduction mechanism. Since OLC15 affected the late, long-lasting pheromone response during the activity phase, we concluded that Orco is slowly activated either voltage-, and/or second messenger-dependently as a consequence of activation of a metabotropic pheromone transduction cascade. To test whether Orco might be activated voltage-dependently, during the pheromone-dependent receptor potential, we performed current injection experiments. Injection-dependent depolarization gated ion channels, which increased spontaneous AP activity, more strongly during the moth´s activity phase than during rest (Fig 9, Table 3). Since OLC15 was able to block depolarization-dependent increases in spontaneous activity of ORNs (Table 3), depolarization opens Orco either directly or indirectly. Thus, we conclude that Orco is a slow, apparently directly voltage-gated ion channel, which is ZT-dependently expressed or modified in ORNs *in vivo*.

**Table 3:** Statistics for current injection experiments.

*P<0.05; **P<0.01; ***P<0.001	P-value			P-value
	Activity	Rest		Activity–Rest
DMSO–DMSO (3 nA)	<0.0001	<0.0001	DMSO	0.0107
OLC15 –OLC15 (3 nA)	0.5025	0.0446	DMSO (3 nA)	0.0005
DMSO–OLC15	0.2663	<0.0001	OLC15	0.9456
DMSO (3 nA)–OLC15 (3 nA)	<0.0001	0.2339	OLC15 (3 nA)	0.0224

Mann-Whitney tests were used for comparison of each pair of experiments; α = 0.05. Concentration of OLC15 in all experiments: 10 μM.

**Fig 10:**
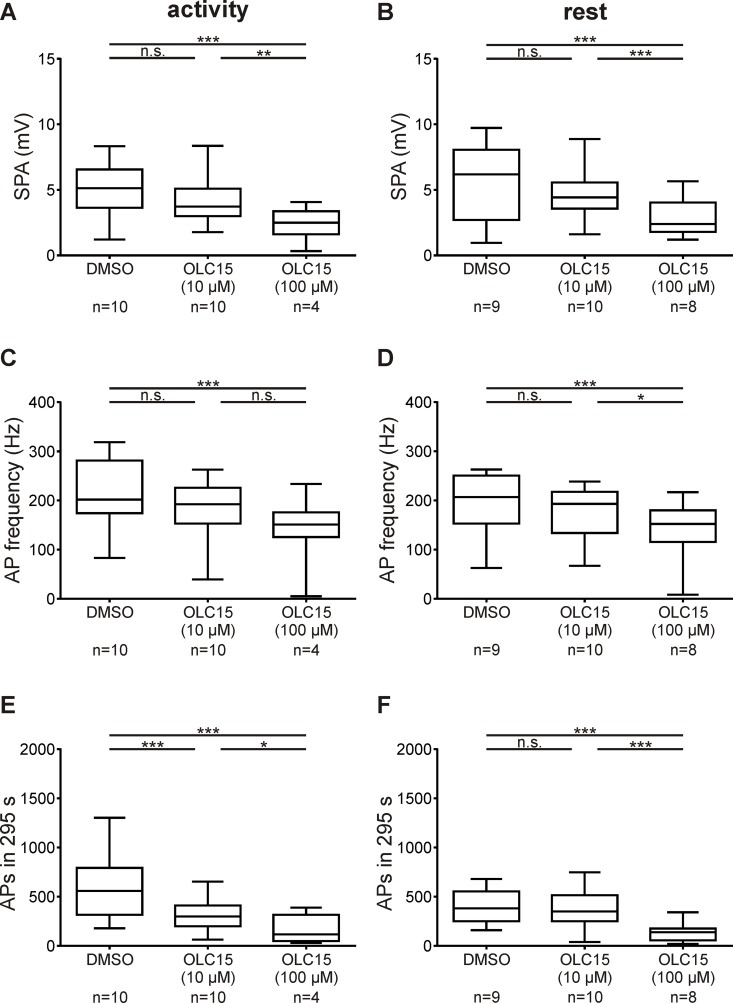
Only at very high concentrations Orco antagonist OLC15 (100 μM) inhibited the immediate pheromone response. While intermediate concentrations (10 μM) of OLC15 did not change the sensillum potential amplitude (SPA) and the frequency of the first 10 bombykal-dependent APs, higher OLC15 concentrations significantly decreased both parameters during activity and rest (A-D). In contrast, intermediate concentrations (10 μM) of OLC15 already strongly reduced the late, long-lasting pheromone response (LLPR) during activity (E). Only higher concentrations were effective at rest, hinting unspecific effects on other targets than Orco (F). Box plots with whiskers from 5 to 95 percentiles. Asterisks indicate significant differences (exact P-values see Table 2; n.s. = not significant; *P<0.05; **P<0.01; ***P<0.001).

If MsexOrco is a voltage-dependent ion channel which opens at negative potentials and depolarizes ORNs keeping them close to spike threshold, very strong block of Orco should hyperpolarize ORNs, finally affecting all phases of the pheromone response. It was previously shown that pheromone as well as IP_3_ first open a transient Ca^2+^ channel [2, 33, 35]. The resulting transient influx of Ca^2+^ started a cascade of directly, and indirectly Ca^2+^-dependent ion channels to open. They appeared to underlie the pheromone-dependent sensillum potential and the phasic AP response [2, 33, 35]. Thus, higher concentrations of OLC15 should decrease the current via this pheromone-dependent Ca^2+^ channel, owing to strong hyperpolarization. Indeed, at higher doses of OLC15 not only the late, long-lasting pheromone response was affected by OLC15, but also the sensillum potential and the phasic pheromone response (Fig 10).

## Discussion

Two studies in different heterologous expression systems providing contradicting results on odor transduction in the fruitfly *Drosophila melanogaster* greatly stimulated the field of insect odor transduction research [17, 18]. Sato et al. [17] proposed a purely ionotropic transduction mechanism based upon OR-Orco receptor ion channel heteromers in all insect species, for general odor detection as well as for pheromone detection [17]. In contrast, Wicher et al. [18] hypothesized that in addition to a fast Orco-dependent ionotropic cascade of low sensitivity, ORs couple to G_αs_ and, thus, support an additional slower, metabotropic transduction cascade of high sensitivity [18].

Many groups took up the challenge to resolve these apparent contradictions and to examine the function of Orco in insect odor transduction. (reviews: [1, 2, 6, 37]). Since OR-expression in heterologous expression systems in the absence of Orco still allowed for odor responses, Orco is not obligatory for odor responses as long as ORs are expressed in the membranes of dendritic cilia (review: [6]). However, *in vivo* studies on intact olfactory sensilla testing Orco function in the animal are rare. Moreover, during analyses no attention was paid to the different kinetic phases of the odor-dependent AP response. Also, no attention was paid to Zeitgebertimes (ZTs), or to odor concentrations tested, despite the fact that odor-sensitivity and odor-dependent behavior express circadian rhythms (review [2]). We, therefore, concentrated on *in vivo* tip-recordings of single pheromone-sensitive trichoid sensilla of the hawkmoth *M*. *sexta*, and dissected fast phasic from late long-lasting pheromone responses (Fig 4A). In addition, we paid attention to ZT-dependent effects and to the doses of pheromone and chemicals employed. Previous *in vitro* patch clamp experiments on primary cell cultures of *M*. *sexta* ORNs suggested a metabotropic odor transduction cascade via G_q_-dependent activation of phospholipase Cβ for detection of both, pheromones, as well as general odors (review: [2]). Rising levels of IP_3_ and DAG triggered a rapid and transient influx of Ca^2+^ via an IP_3_-dependent Ca^2+^ channel. Then, intracellular Ca^2+^ rises opened a specific sequence of Ca^2+^-dependently and protein kinase C (PKC)-dependently gated cation channels. These channel openings appeared to underlie the phasic and tonic pheromone responses [2, 33, 35]. The IP_3_- and Ca^2+^-dependent currents were measured in almost all ORNs in primary cell cultures that contained only ~37% of pheromone-sensitive ORNs, while the other ORNs responded to general odorants [33, 35]. Thus, we assume that PLCβ-dependent transduction cascades are employed both, for detection of general odors, and for detection of pheromones. At higher pheromone concentrations, or prolonged pheromone stimulation, G-protein-independent receptor guanylyl cyclases were activated, which increased intracellular cGMP levels [38–40]. These cGMP rises then activated cGMP-dependent ion channels which opened during the time window of the late, long-lasting pheromone response [41, 42]. Biochemical measurements confirmed pheromone-dependent activation of phospholipase Cβ. They demonstrated increases in IP_3_ levels within the first 100 ms of the pheromone response and a later, delayed activation of guanylyl cyclases, but not adenylyl cyclases in moths and cockroaches [39, 43]. In contrast to pheromone the stress hormone octopamine increased cAMP levels in hawkmoth and cockroach antennae [44].

The amiloride-dependent decrease of the bombykal-dependent sensillum potential and the phasic AP response reported here are consistent with this hypothesis of metabotropic pheromone transduction, because amiloride blocks Ca^2+^-dependent cation channels in moth ORNs. These channels resemble bombykal- and PLCβ-dependent channels in the hawkmoth [2, 26, 33, 35]. The PKC-dependent currents predominate pheromone responses during rest, in contrast to Ca^2+^-dependent cation currents, which predominate during activity [2]. Thus, current studies examine, whether MIA more specifically blocks the Ca^2+^-dependent cation channel, while HMA more specifically blocks PKC-dependent ion channels. In addition, we hypothesize that these different ion channels involved in pheromone transduction change ZT-dependently in abundance or gating properties. Altogether, our findings demonstrate that there are at least three different ion channels involved in the pheromone transduction cascade that open at specific time windows of the pheromone response and that are differentially sensitive to both amiloride derivatives and to OLC15. In addition, all three ion channels are expressed ZT-dependently. Thus, in contrast to the suggestion by Pask et al. [27], both amiloride derivatives do not primarily target Orco, since OLC15, MIA, and HMA affected different targets [27]. However, it cannot be excluded that amilorides additionally also affected Orco. Despite the fact that we found in our experiments (not shown) that amiloride derivatives block VUAA1-dependent activity, we cannot discern, whether they blocked Orco in addition to Ca^2+^ gated currents. Orco opening will always elevate intracellular Ca^2+^ concentrations which, in turn, activate different Ca^2+^-gated ion channels described in hawkmoth ORNs ([2, 26, 33, 35]). Also in the experiments performed by Pask et al. [27] it remains to be examined, whether in the heterologous expression systems the Orco-dependent influx of Ca^2+^ activated Ca^2+^-dependent cation channels that were targeted by the amiloride-derivatives instead of, or in addition to Orco.

In a comparison of VUAA1 actions of MsexOrco in heterologous expression to VUAA1 actions in primary cell cultures of hawkmoth ORNs, we provided evidence that Orco-activator VUAA1 also targets MsexOrco in its natural environment in the sensory neurons. Western blots confirmed that MsexOrco is expressed in pupal and adult antennae during the time windows we observed VUAA1-effects. They also indicated that Orco plays a role during development of the antennal ORNs which is independent of ORs. Because Orco mutations deleted spontaneous activity, there is general consensus that Orco is a pacemaker channel which controls spontaneous activity of ORNs *in vivo* [6, 15, 19, 20]. By increasing the spontaneous activity in intact, adult antennae VUAA1-dependently and because of its dose-dependent block by OLC15, we provided further evidence that both agents target Orco *in vivo* [19, 23]. Thus, we successfully determined intermediate concentrations of OLC15 obtaining about half maximal block of MsexOrco *in vivo* as prerequisite to a distinction of MsexOrco functions as pacemaker channel or as receptor ion channel complex.

Because activation of MsexOrco via VUAA1 [19] as well as inactivation of MsexOrco via OLC15 did not affect the sensillum potential amplitude at intermediate concentrations, it is highly unlikely that odor-dependent opening of OR-Orco heteromers is the primary pheromone transduction event. Consistently, MsexOrco inhibition with intermediate concentrations of OLC15 or VUAA1-dependent activation of MsexOrco did not affect the frequency of the phasic AP response, which occurs within less than 100 ms of the odor response [19]. Instead, MsexOrco-inhibition at intermediate concentrations significantly decreased the spontaneous AP frequency and also prolonged the latency of the first, pheromone-dependent AP. Therefore, MsexOrco controls the membrane potential and, thereby, the AP threshold of ORNs, as is typical for a pacemaker channel. Because hawkmoth ORNs are spontaneously active and interspike intervals of their spontaneous activity are not distributed randomly, ORNs express regular membrane potential oscillations [31, 45]. Since MsexOrco strongly affects spontaneous activity, and since it opens spontaneously in heterologous expression systems, it appears to act as a pacemaker channel that opens at rest, causing subthreshold membrane potential oscillations. Intracellular recordings from primary cultures of ORNs showed that pheromone stimulation strongly affected membrane potential oscillations of ORNs [46]. Therefore, in addition to being circadian pacemakers, ORNs are ultradian oscillators which can resonate easily, if stimulated with pulse trains [2, 47]. This property might be responsible for the amazingly rapid kinetics of insect antennae, measured recently [48]. Increasing concentrations of MsexOrco inhibition increasingly hyperpolarized ORNs and lead them further away from spike threshold. Finally, this increasing hyperpolarization would also decrease the sensillum potential amplitude and the phasic pheromone response. It would strongly affect kinetics and threshold of odor responses, as shown here. Consequently, at high concentrations of Orco antagonists and agonists it is not possible any more, to distinguish between a function of Orco as pacemaker channel, which controls the membrane potential, or a function of Orco in ionotropic signal transduction. Therefore, to distinguish between both functions of Orco, blocker experiments should not employ concentrations of the antagonist which are higher than ~IC_50_ values.

**Fig 11:**
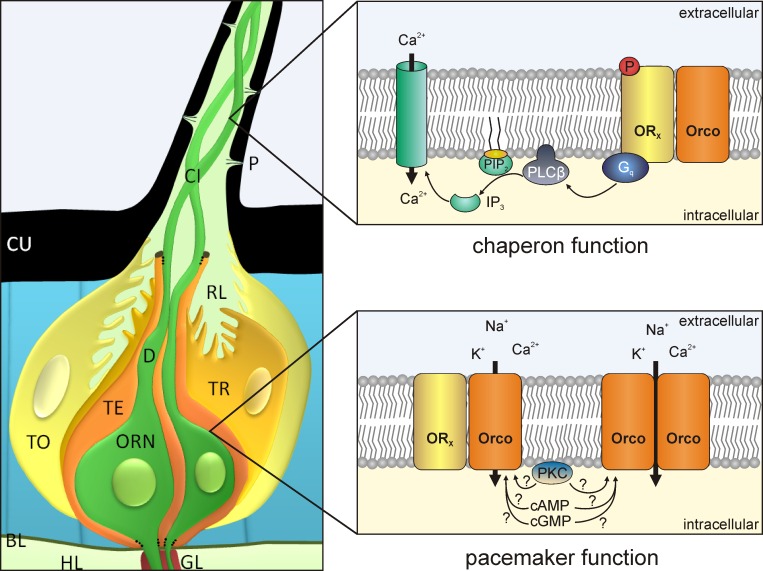
Scheme of Orco functions in hawkmoth olfactory sensilla (modified after review: [2]). MsexOrco is suggested to play no role for the primary events of odor/pheromone transduction. In the dendritic cilia Orco acts as “chaperon”, locating and maintaining odor-binding olfactory receptors (OR_x_) in membranes of cilia, possibly Ca^2+^/calmoduline-dependently, as shown recently [49]. Odor/pheromone binding to ORs activates a phospholipase (PLCβ) that hydrolyzes phospholipids (PIP_2_). The metabotropic odor/pheromone transduction generates rises in IP_3_ which open first a Ca^2+^ channel. The rise in intracellular Ca^2+^ starts a cascade of ion channel openings (not shown) that generate the depolarizing receptor potential. The resulting odor-dependent voltage-changes gate Orco in the soma of the olfactory receptor neurons. There, MsexOrco acts as pacemaker channel that controls membrane potential oscillations and, thus, spike threshold and response kinetics. It remains to be studied whether MsexOrco is also gated via cAMP and cGMP, or via protein kinase C-dependent phosphorylation. BL basal lamina, CI cilium, CU cuticle, D dendrite, GL glia, HL hemolymph, ORN olfactory receptor neuron, P pore, RE receptor lymph, TE thecogen cell, TO tormogen cell, TR trichogen cell.

How and when is Orco activated during pheromone transduction? Our current injection experiments demonstrated that MsexOrco is activated by depolarization directly or indirectly. Because MsexOrco was not opened during the first 100 ms of the rising sensillum potential, but rather several hundred ms to minutes after pheromone stimulation, only MsexOrco inserted into the soma membrane, may possibly contribute to odor-dependent potential changes. This would suggest distinct functions for Orco, depending on its location and possibly, depending on homo- or hetero-multimerization (Fig 11). Additionally, Orco in its *in vivo* environment is a slow ion channel. Possibly, it needs to be phosphorylated or otherwise modified via the pheromone-dependent second messenger increases of the metabotropic pheromone transduction cascade, before it can open. The last hypothesis is supported by findings in *D*. *melanogaster*. Fruitfly Orco has several phosphorylation sites and needs to be phosphorylated first via PKC, before it opens via binding cGMP or cAMP [21, 22]. The delayed rises in cGMP levels in moth antennae might then keep Orco activated over several minutes [38–40]. Future experiments will test whether also in other insect species Orco is not involved in ionotropic, but rather in metabotropic and also in G-protein-independent guanylyl cyclase-dependent odor transduction cascades.

## Supporting Information

S1 Table Mean ± std. error of spontaneous activity in the presence or absence of 10 μM VUAA1 in combination with different concentrations of OLC15. (DOCX)

S2 Table Mean values ± std. error of the response parameters in control recordings and under the influence of MIA, HMA or OLC15 (10 μM, each). (DOCX)

S3 Table Mean ± std. error for current injection experiments in the presence and absence of 10 μM OLC15 (DOCX)

S4 Table Mean values ± std. error of higher OLC15 concentrations (100 μM). (DOCX)

## References

[1] Nakagawa T, Vosshall LB. Controversy and consensus: noncanonical signaling mechanisms in the insect olfactory system. Curr Opin Neurobiol. 2009 6;19(3):284–92. 10.1016/j.conb.2009.07.015 19660933 PMC2752668

[2] Stengl M. Pheromone transduction in moths. Front Cell Neurosci. 2010;4:133 10.3389/fncel.2010.00133 21228914 PMC3018772

[3] Benton R, Sachse S, Michnick SW, Vosshall LB. Atypical membrane topology and heteromeric function of *Drosophila* odorant receptors in vivo. PLoS Biol. 2006 2;4(2):e20 10.1371/journal.pbio.0040020 16402857 PMC1334387

[4] Wistrand M, Kall L, Sonnhammer EL. A general model of G protein-coupled receptor sequences and its application to detect remote homologs. Protein Sci. 2006 3;15(3):509–21. 10.1110/ps.051745906 16452613 PMC2249772

[5] Lundin C, Kall L, Kreher SA, Kapp K, Sonnhammer EL, Carlson JR, et al Membrane topology of the *Drosophila* OR83b odorant receptor. FEBS Lett. 2007 12 11;581(29):5601–4. 10.1016/j.febslet.2007.11.007 18005664 PMC2176074

[6] Stengl M, Funk NW. The role of the coreceptor Orco in insect olfactory transduction. J Comp Physiol A Neuroethol Sens Neural Behav Physiol. 2013 11;199(11):897–909. 10.1007/s00359-013-0837-3 23824225

[7] Clyne PJ, Warr CG, Freeman MR, Lessing D, Kim J, Carlson JR. A novel family of divergent seven-transmembrane proteins: candidate odorant receptors in *Drosophila*. Neuron. 1999 2;22(2):327–38. 10069338 10.1016/s0896-6273(00)81093-4

[8] Vosshall LB, Amrein H, Morozov PS, Rzhetsky A, Axel R. A spatial map of olfactory receptor expression in the *Drosophila* antenna. Cell. 1999 3 5;96(5):725–36. 10089887 10.1016/s0092-8674(00)80582-6

[9] Krieger J, Raming K, Dewer YM, Bette S, Conzelmann S, Breer H. A divergent gene family encoding candidate olfactory receptors of the moth *Heliothis virescens*. Eur J Neurosci. 2002 8;16(4):619–28. 12270037 10.1046/j.1460-9568.2002.02109.x

[10] Krieger J, Klink O, Mohl C, Raming K, Breer H. A candidate olfactory receptor subtype highly conserved across different insect orders. J Comp Physiol A Neuroethol Sens Neural Behav Physiol. 2003 7;189(7):519–26. 10.1007/s00359-003-0427-x 12827420

[11] Elmore T, Ignell R, Carlson JR, Smith DP. Targeted mutation of a *Drosophila* odor receptor defines receptor requirement in a novel class of sensillum. J Neurosci. 2003 10 29;23(30):9906–12. 14586020 10.1523/JNEUROSCI.23-30-09906.2003 PMC6740877

[12] Hallem EA, Carlson JR. The odor coding system of *Drosophila*. Trends Genet. 2004 9;20(9):453–9. 10.1016/j.tig.2004.06.015 15313555

[13] Dobritsa AA, van der Goes van Naters W, Warr CG, Steinbrecht RA, Carlson JR. Integrating the molecular and cellular basis of odor coding in the *Drosophila* antenna. Neuron. 2003 3 6;37(5):827–41. 12628173 10.1016/s0896-6273(03)00094-1

[14] Hallem EA, Ho MG, Carlson JR. The molecular basis of odor coding in the *Drosophila* antenna. Cell. 2004 6 25;117(7):965–79. 10.1016/j.cell.2004.05.012 15210116

[15] Larsson MC, Domingos AI, Jones WD, Chiappe ME, Amrein H, Vosshall LB. Or83b encodes a broadly expressed odorant receptor essential for *Drosophila* olfaction. Neuron. 2004 9 2;43(5):703–14. 10.1016/j.neuron.2004.08.019 15339651

[16] Neuhaus EM, Gisselmann G, Zhang W, Dooley R, Stortkuhl K, Hatt H. Odorant receptor heterodimerization in the olfactory system of *Drosophila melanogaster*. Nat Neurosci. 2005 1;8(1):15–7. 10.1038/nn1371 15592462

[17] Sato K, Pellegrino M, Nakagawa T, Vosshall LB, Touhara K. Insect olfactory receptors are heteromeric ligand-gated ion channels. Nature. 2008;452(7190):1002–06. 10.1038/nature06850 18408712

[18] Wicher D, Schafer R, Bauernfeind R, Stensmyr MC, Heller R, Heinemann SH, et al *Drosophila* odorant receptors are both ligand-gated and cyclic-nucleotide-activated cation channels. Nature. 2008 4 24;452(7190):1007–11. 10.1038/nature06861 18408711

[19] Nolte A, Funk NW, Mukunda L, Gawalek P, Werckenthin A, Hansson BS, et al In situ tip-recordings found no evidence for an Orco-based ionotropic mechanism of pheromone-transduction in *Manduca sexta*. PLoS One. 2013;8(5):e62648 10.1371/journal.pone.0062648 23671617 PMC3643954

[20] Benton R. Sensitivity and specificity in *Drosophila* pheromone perception. Trends Neurosci. 2007 10;30(10):512–9. 10.1016/j.tins.2007.07.004 17825436

[21] Sargsyan V, Getahun MN, Llanos SL, Olsson SB, Hansson BS, Wicher D. Phosphorylation via PKC regulates the function of the *Drosophila* odorant co-receptor. Front Cell Neurosci. 2011;5:5 10.3389/fncel.2011.00005 21720521 PMC3118453

[22] Getahun MN, Olsson SB, Lavista-Llanos S, Hansson BS, Wicher D. Insect odorant response sensitivity is tuned by metabotropically autoregulated olfactory receptors. PLoS One. 2013;8(3):e58889 10.1371/journal.pone.0058889 23554952 PMC3595248

[23] Jones PL, Pask GM, Rinker DC, Zwiebel LJ. Functional agonism of insect odorant receptor ion channels. Proc Natl Acad Sci U S A. 2011 5 24;108(21):8821–5. 10.1073/pnas.1102425108 21555561 PMC3102409

[24] Chen SS, Luetje CW. Identification of new agonists and antagonists of the insect odorant receptor co-receptor subunit. PLoS One. 2012 5 8;7(5):e36784 10.1371/journal.pone.0036784 22590607 PMC3348135

[25] Bobkov YV, Ache BW. Block by amiloride derivatives of odor-evoked discharge in lobster olfactory receptor neurons through action on a presumptive TRP channel. Chem Senses. 2007 2;32(2):149–59. 10.1093/chemse/bjl041 17098814

[26] Zufall F, Hatt H. A calcium-activated nonspecific cation channel from olfactory receptor neurones of the silkmoth *Antheraea polyphemus*. J Exp Biol. 1991 11;161:455–68. 22141156 10.1242/jeb.161.1.455

[27] Pask GM, Bobkov YV, Corey EA, Ache BW, Zwiebel LJ. Blockade of insect odorant receptor currents by amiloride derivatives. Chem Senses. 2013 3;38(3):221–9. 10.1093/chemse/bjs100 23292750 PMC3569625

[28] Martin JP, Beyerlein A, Dacks AM, Reisenman CE, Riffell JA, Lei H, et al The neurobiology of insect olfaction: sensory processing in a comparative context. Prog Neurobiol. 2011 11;95(3):427–47. 10.1016/j.pneurobio.2011.09.007 21963552

[29] Hildebrand JG, Shepherd GM. Mechanisms of olfactory discrimination: converging evidence for common principles across phyla. Annu Rev Neurosci. 1997;20:595–631. 10.1146/annurev.neuro.20.1.595 9056726

[30] Flecke C, Nolte A, Stengl M. Perfusion with cAMP analogue affects pheromone-sensitive trichoid sensilla of the hawkmoth *Manduca sexta* in a time-dependent manner. J Exp Biol. 2010 3 1;213(5):842–52. 10.1242/jeb.032839 20154200

[31] Dolzer J, Fischer K, Stengl M. Adaptation in pheromone-sensitive trichoid sensilla of the hawkmoth *Manduca sexta*. J Exp Biol. 2003 5;206(Pt 9):1575–88. 12654896 10.1242/jeb.00302

[32] Stengl M, Hildebrand JG. Insect olfactory neurons in vitro: morphological and immunocytochemical characterization of male-specific antennal receptor cells from developing antennae of male *Manduca sexta*. J Neurosci. 1990 3;10(3):837–47. 2319305 10.1523/JNEUROSCI.10-03-00837.1990 PMC6570139

[33] Stengl M. Intracellular-messenger-mediated cation channels in cultured olfactory receptor neurons. J Exp Biol. 1993 5;178:125–47. 7686208 10.1242/jeb.178.1.125

[34] Wei H, Yasar H, Funk NW, Giese M, Baz E-S, Stengl M. Signaling of pigment-dispersing factor (PDF) in the Madeira cockroach *Rhyparobia maderae*. PLoS One. 2014;9(9):e108757 10.1371/journal.pone.0108757 25269074 PMC4182629

[35] Stengl M. Inositol-trisphosphate-dependent calcium currents precede cation currents in insect olfactory receptor neurons in vitro. J Comp Physiol A. 1994 2;174(2):187–94. 7511689 10.1007/BF00193785

[36] Schweitzer ES, Sanes JR, Hildebrand JG. Ontogeny of electroantennogram responses in the moth, *Manduca sexta*. J Insect Physiol. 1976;22(7):955–60. 947988 10.1016/0022-1910(76)90078-0

[37] Touhara K, Vosshall LB. Sensing odorants and pheromones with chemosensory receptors. Annu Rev Physiol. 2009;71:307–32. 10.1146/annurev.physiol.010908.163209 19575682

[38] Ziegelberger G, van den Berg MJ, Kaissling KE, Klumpp S, Schultz JE. Cyclic GMP levels and guanylate cyclase activity in pheromone-sensitive antennae of the silkmoths *Antheraea polyphemus* and *Bombyx mori*. J Neurosci. 1990 4;10(4):1217–25. 1970356 10.1523/JNEUROSCI.10-04-01217.1990 PMC6570213

[39] Boekhoff I, Seifert E, Goggerle S, Lindemann M, Kruger BW, Breer H. Pheromone-induced second-messenger signaling in insect antennae. Insect Biochem Molec. 1993 10;23(7):757–62.

[40] Stengl M, Zintl R, De Vente J, Nighorn A. Localization of cGMP immunoreactivity and of soluble guanylyl cyclase in antennal sensilla of the hawkmoth *Manduca sexta*. Cell Tissue Res. 2001 6;304(3):409–21. 11456418 10.1007/s004410000336

[41] Dolzer J, Krannich S, Stengl M. Pharmacological investigation of protein kinase C- and cGMP-dependent ion channels in cultured olfactory receptor neurons of the hawkmoth *Manduca sexta*. Chem Senses. 2008 11;33(9):803–13. 10.1093/chemse/bjn043 18635555 PMC2580732

[42] Krannich S, Stengl M. Cyclic nucleotide-activated currents in cultured olfactory receptor neurons of the hawkmoth *Manduca sexta*. J Neurophysiol. 2008 11;100(5):2866–77. 10.1152/jn.01400.2007 18684910

[43] Breer H, Boekhoff I, Tareilus E. Rapid kinetics of second messenger formation in olfactory transduction. Nature. 1990 5 3;345(6270):65–8. 10.1038/345065a0 2158631

[44] Schendzielorz T, Schirmer K, Stolte P, Stengl M. Octopamine regulates antennal sensory neurons via daytime-dependent changes in cAMP and IP3 levels in the hawkmoth *Manduca sexta*. PLoS One. 2015;10(3):e0121230 10.1371/journal.pone.0121230 25785721 PMC4364694

[45] Dolzer J, Krannich S, Fischer K, Stengl M. Oscillations of the transepithelial potential of moth olfactory sensilla are influenced by octopamine and serotonin. J Exp Biol. 2001 8;204(Pt 16):2781–94. 11683434 10.1242/jeb.204.16.2781

[46] Stengl M. Development of an in vitro model system to study primary sensory transduction mechanisms [PhD-thesis]. Tucson: University of Arizona; 1990.

[47] Schuckel J, Siwicki KK, Stengl M. Putative circadian pacemaker cells in the antenna of the hawkmoth *Manduca sexta*. Cell Tissue Res. 2007 11;330(2):271–8. 10.1007/s00441-007-0471-x 17786482

[48] Szyszka P, Gerkin RC, Galizia CG, Smith BH. High-speed odor transduction and pulse tracking by insect olfactory receptor neurons. Proc Natl Acad Sci U S A. 2014 11 25;111(47):16925–30. 10.1073/pnas.1412051111 25385618 PMC4250155

[49] Bahk S, Jones WD. Insect odorant receptor trafficking requires calmodulin. BMC Biol. 2016;14:83 10.1186/s12915-016-0306-x 27686128 PMC5043534

