# Supplementary material for: No Evidence for Ionotropic Pheromone Transduction in the Hawkmoth Manduca sexta
Source: PLoS One. 2016 Nov 9;11(11):e0166060. doi: 10.1371/journal.pone.0166060 (PMC5102459; doi:10.1371/journal.pone.0166060)
Supplement: S1 Table — (DOCX) [file pone.0166060.s001.docx]

S1 Table. Mean values of spontaneous activity (Fig 3) in the presence or absence of 10 µM VUAA1 in combination with different concentrations of OLC15.

|  | DMSO n=14 | 10 µM VUAA1 n=9 | VUAA1 + 1 µM OLC15 n=10 | VUAA1 + 10 µM OLC15 n=12 | VUAA1 + 100 µM OLC15 n=9 |
| --- | --- | --- | --- | --- | --- |
| Resting phase (ZT 9) | 26.21 ± 9.32 | 398.6 ± 41.24 | 332.9 ± 25.49 | 248.6 ± 20.30 | 148.7 ± 14.10 |
|  | **DMSO n=16** | **10 µM VUAA1 n=12** | **VUAA1 + 1 µM OLC15 n=9** | **VUAA1 + 10 µM OLC15 n=14** | **VUAA1 + 100 µM OLC15 n=12** |
| Late activity phase (ZT 1) | 41.16 ± 5.16 | 591.0 ± 55.47 | 389.9 ± 29.36 | 326.8 ± 24.64 | 168.0 ± 17.12 |

Values are given as number of action potentials in 295 s (mean ± std. error).
