# Supplementary material for: No Evidence for Ionotropic Pheromone Transduction in the Hawkmoth Manduca sexta
Source: PLoS One. 2016 Nov 9;11(11):e0166060. doi: 10.1371/journal.pone.0166060 (PMC5102459; doi:10.1371/journal.pone.0166060)
Supplement: S2 Table — (DOCX) [file pone.0166060.s002.docx]

S2 Table. Mean values ± std. error of the response parameters in control recordings and under the influence of MIA, HMA or OLC15 (10 µM, each).

|  | | norm. SPA | latency (ms) | APF (Hz) | LLPR |
| --- | --- | --- | --- | --- | --- |
| Resting phase  (ZT 9) | DMSO n=9 | 0.992 ± 0.033 | 16.21 ± 2.14 | 197.7 ± 9.16 | 399.9 ± 25.14 |
|  | 10 µM MIA n=10 | 0.751 ± 0.046 | 60.07 ± 19.77 | 159.9 ± 11.41 | 383.2 ± 32.93 |
|  | 10 µM HMA n=10 | 0.659 ± 0.046 | 114.3 ± 28.32 | 98.42 ± 11.13 | 182.1 ± 26.83 |
|  | 10 µM OLC15 n=10 | 0.984 ± 0.037 | 43.36 ± 13.57 | 174.1 ± 7.74 | 379.5 ± 28.91 |
| Activity phase  (ZT 1) | DMSO n=10 | 0.966 ± 0.035 | 18.75 ± 1.92 | 220.8 ± 11.27 | 604.3 ± 47.94 |
|  | 10 µM MIA n=10 | 0.772 ± 0.056 | 98.46 ± 37.63 | 151.2 ± 12.15 | 293.8 ± 34.45 |
|  | 10 µM HMA n=10 | 0.792 ± 0.035 | 123.8 ± 44.72 | 171.6 ± 17.14 | 326.0 ± 32.58 |
|  | 10 µM OLC15 n=10 | 1.125 ± 0.073 | 90.03 ± 39.38 | 181.2 ± 9.05 | 311.9 ± 22.66 |

Values for late, long-lasting pheromone response (LLPR) are given as number of action potentials in 295 s
