# Supplementary material for: No Evidence for Ionotropic Pheromone Transduction in the Hawkmoth Manduca sexta
Source: PLoS One. 2016 Nov 9;11(11):e0166060. doi: 10.1371/journal.pone.0166060 (PMC5102459; doi:10.1371/journal.pone.0166060)
Supplement: S3 Table — (DOCX) [file pone.0166060.s003.docx]

S3 Table. Mean ± std. error for current injection experiments in the presence and absence of 10 µM OLC15

|  | DMSO, 0 nA n=18 | | DMSO, 3 nA n=12 | 10 µM OLC15,  0 nA n=10 | 10 µM OLC15,  3 nA n=17 |
| --- | --- | --- | --- | --- | --- |
| Resting phase (ZT 9) | | 12.57 ± 2.61 | 53.50 ± 8.13 | 21.31 ± 3.72 | 33.68 ± 3.83 |
|  | | **DMSO, 0 nA n=18** | **DMSO, 3 nA n=12** | **10 µM OLC15,  0 nA n=10** | **10 µM OLC15,  3 nA n=11** |
| Activity phase (ZT 1) | | 16.21 ± 2.42 | 92.47 ± 8.33 | 22.87 ± 5.44 | 22.95 ± 4.72 |

Values are given as number of action potentials in 60 s
