# Supplementary material for: No Evidence for Ionotropic Pheromone Transduction in the Hawkmoth Manduca sexta
Source: PLoS One. 2016 Nov 9;11(11):e0166060. doi: 10.1371/journal.pone.0166060 (PMC5102459; doi:10.1371/journal.pone.0166060)
Supplement: S4 Table — (DOCX) [file pone.0166060.s004.docx]

S4 Table. Mean values ± std. error of higher OLC15 concentrations (100 µM).

| Resting phase (ZT 9) | SPA (mV) | APF (Hz) | LLPR |
| --- | --- | --- | --- |
| DMSO n=10 | 5.362 ± 0.457 | 197.7 ± 9.16 | 399.9 ± 25.14 |
| 10 µM OLC15 n=10 | 4.739 ± 0.277 | 174.1 ± 7.74 | 379.5 ± 28.91 |
| 100 µM OLC15 n=8 | 2.831 ± 0.196 | 138.7 ± 9.08 | 136.4 ± 14.43 |
| Activity phase (ZT 1) | | | |
| DMSO n=10 | 5.022 ± 0.313 | 220.8 ± 11.27 | 604.3 ± 47.94 |
| 10 µM OLC15 n=10 | 4.164 ± 0.260 | 181.2 ± 9.05 | 311.9 ± 22.66 |
| 100 µM OLC15 n=4 | 2.487 ± 0.243 | 147.9 ± 13.80 | 169.7 ± 29.94 |

Values for late, long-lasting pheromone response (LLPR) are given as number of action potentials in 295 s
